# Introducing the Novel Mixed Gaussian-Lorentzian Lineshape in the Analysis of the Raman Signal of Biochar

**DOI:** 10.3390/nano10091748

**Published:** 2020-09-03

**Authors:** Alberto Tagliaferro, Massimo Rovere, Elisa Padovano, Mattia Bartoli, Mauro Giorcelli

**Affiliations:** 1 Department of Applied Science and Technology, Politecnico di Torino, Corso Duca degli Abruzzi 24, 10129 Turin, Italy; massimo.rovere@polito.it (M.R.); elisa.padovano@polito.it (E.P.); mattia.bartoli@polito.it (M.B.); mauro.giorcelli@polito.it (M.G.); 2 Faculty of Science, Ontario Tech University, 2000 Simcoe Street North, Oshawa, ON L1G 0C5 T, Canada; 3 Consorzio Interuniversitario Nazionale per la Scienza e Tecnologia dei Materiali (INSTM), Via G. Giusti 9, 50121 Florence, Italy

**Keywords:** Raman, disordered carbon, biochar, signal processing

## Abstract

In this research, an innovative procedure is proposed to elaborate Raman spectra obtained from nanostructured and disordered solids. As a challenging case study, biochar, a bio-derived carbon based material, was selected. The complex structure of biochar (i.e., channeled surface, inorganic content) represents a serious challenge for Raman characterization. As widely reported, the Raman spectra are closely linked to thermal treatments of carbon material. The individual contributions to the Raman spectra are difficult to identify due to the numerous peaks that contribute to the spectra. To tackle this problem, we propose a brand new approach based on the introduction, on sound theoretical grounds, of a mixed Gaussian-–Lorentzian lineshape. As per the experimental part, biochar samples were carbonized in an inert atmosphere at various temperatures and their respective spectra were successfully decomposed using the new lineshape. The evolution of the structure with carbonization temperature was investigated by Raman and XRD analysis. The results of the two techniques fairly well agree. Compared to other approaches commonly reported in the literature this method (i) gives a sounder basis to the lineshape used in disordered materials, and (ii) appears to reduce the number of components, leading to an easier understanding of their origin.

## 1. Introduction

From the beginning of human history, carbon materials have played a crucial role in human life. In more recent times, new forms of carbon such as fullerene, carbon nanotubes and graphene have become widely available. These new forms have provided new perspectives of application to carbon materials. As the structural quality of a material is often critical to determine its properties, the structural investigation of carbon materials is of paramount importance. Among the characterization techniques used to investigate the local nanostructure of carbon materials Raman microscopy is the most widespread [1]. Raman spectroscopy offers a couple of interesting advantages. On the one hand it is a non destructive technique and as such can also be placed online as a quality control tool in production systems. On the other hand, each ordered carbon material has a distinctive Raman spectrum that constitutes its fingerprint [2] and relevant information can be additionally extracted from the spectra. Two specific spectral ranges are of interest for sp^2^ coordinated carbon materials, which will form the basis for later discussion: (i) the region in the range 1000 to 2000 cm^−1^ where the main peaks are D and G [2] seldom accompanied by less intense peaks, and (ii) the range 2400 to 3300 cm^−1^ where the overtones of the D and G peaks give their contribution [3]. In highly crystalline ordered materials the peaks are sharp and well separated so that the identification of the peaks and their quantification in terms of intensity, centers, and linewidth is straightforward, and their lineshape can be approximated to a Lorentzian curve [4]. However, when the carbon materials are disordered things become more complicated and blurred as the width of the peaks increases, and neighboring peaks start to merge. In the extreme situation of diamond like carbon, featureless bumps appear in both ranges and the identification of the specific components becomes rather challenging [5]. Lorentzian curves can also be used for carbon materials which are mainly sp^2^ coordinated, but in which the D and G peaks overlap to form a featured single signal in which two peaks are evident. Among the various carbon materials Biochar, a charcoal obtained by pyrolysis of vegetal feedstock in an oxygen-poor environment [6], is an optimum case study for our work, since its structure evolves with carbonization temperature and it is far more complex than carbon films or carbon soot due the massively presence of inorganics. As reported by Franklin [7], graphitization process is affected by the mobility of crystallites during the thermal treatment and it is influenced by C/H and C/O ratio of the material. During the years, the carbonization process has been modelized by using several approached reaching the actual distorted graphene triad proposed by McDonald-Wharry et al. [8]. Due to inorganic content and high oxygen content, biochar could be classified into non-graphitizable carbon according to Franklin definition but by increasing pyrolysis or carbonization temperature, biochar undergoes a turbostratic rearrangement with massive formation of sp^2^ cluster. Those domains are composed by randomly distributed disordered and nanographitic domains that can reached up to 80 nm in size and eventually evolve to graphite-like materials for temperature above 2000 °C as in the case of carbonization of cellulose nanocrystals [9].

A number of attempts have been made to decompose the 1000–2000 cm^−1^ range peak into its components [10,11]. To the best of our knowledge, attempts reported in the literature used two components with Gaussian lineshapes. Only Ghosh et al. [12] approached the problem by using a set of Gaussian and Lorentzian curves for Raman spectrum decomposition. However, they did not provide a physical basis for their choice. By fixing the constraint that the lineshape of all peaks should be Gaussian, the number of individual peaks needed to reasonably fit biochar Raman spectra turned out to be quite large [13]. Several authors used Voigt [14] and pseudo-Voigt [15,16] functions to take into account the presence of disordered nanographitic domains. Despite being basically a mix of Lorentzian and Gaussian, in their case the mixing occurs over the whole range of the signal, amounting to assume that two different types of regions (one more ordered, one more disordered) are present in the material, an assumption often not supported by other information. Even if nanographitic domains were identified by microscopical techniques, related Raman signals processing remain largely arbitrary [17]. Therefore, a problem in correlating each peak with a meaningful vibrating mode subsequently arises also in the Voigt and pseudo-Voigt case. Other attempts have been made in this direction; one of the most sophisticated studies is based on first principles computational simulation [18]. However, computation was made for ordered systems and no support from microscopy or other structural investigations was given. In summary, the very complex nature of nanographitic domains and local structure of biochar lead to the problem of properly decomposing Raman spectra being still an open challenge.

In the present paper we will show that by relaxing, on strong physical bases, the constraint of having a Gaussian lineshape, biochar Raman spectra can be decomposed by using two main peaks that can be readily identified as the usual D and G contributions. In order to verify that this approach is consistent we have taken a standardized biochar, produced by pyrolysis at 550 °C, and carbonized it at various temperatures in the range 900–2200 °C. The Raman spectra of all samples were recorded using a laser line at 514 nm. All spectra were satisfactorily decomposed with a single set of peaks. This result was obtained by introducing a lineshape in which three wavenumber ranges are identified for each peak. The central (i.e., closer to the peak highest value) region is fitted with a Lorentzian lineshape, while the two lateral regions are fitted with a Gaussian lineshape. The two lineshapes are matched at the range boundaries providing a unique lineshape for each component used. A thorough discussion in support of this assumption is given the text.

## 2. Materials and Methods

### 2.1. Materials

In order to test our new approach, we selected a standard biochar produced by UK biochar research centre (Edinburg, UK) using a rotary kiln pyrolytic unit [19]. The biochar was produced using oil seed rape (OSR 550) at a pyrolytic temperature of 550 °C [20].

In Table 1 we reported the main physiochemical characteristic of OSR 550.

OSR 550 was further investigated by using a field emission scanning electrical microscope (FE-SEM, Zeis SupraTM40, Oberkochen, Germany) equipped with an energy dispersive X-ray detector (EDX, Oxford Inca Energy 450, Oberkochen, Germany) that was used to explore the inorganic composition of OSR 550 as reported in Table 2.

OSR 550 was investigated in three different sites two of them (Site 1 and Site 2) rich in carbon and one rich in inorganic.

The biochar was further thermally treated (see next paragraph for details) in order to modify the structure from a disordered one to a well ordered one as reported by Lehman [22] so that our approach had to withstand a more tough challenge.

### 2.2. Methods

OSR 550 biochar was subjected to further thermal treatment at temperatures of 900 °C, 1100 °C, 1300 °C, 1500 °C and 2200 °C in order to modify the local structure of the material from disordered to ordered. A vacuum electric furnace from Pro.Ba., Cambiano, Italy was used to carry out the treatments at 900, 1100, 1300 and 1500 °C; the treatment at the highest temperature (2200 °C) was performed using a 448 TAV Vacuum Furnaces oven, Petroceramics, Stezzano, Italy. All treatments were performed under argon atmosphere (99.99% purity, controlled pressure 550 mbar) using a heating rate of 150 °C/h, a dwell at the maximum temperature for 30 min and a cooling to room temperature with the same thermal gradient used for heating. Samples labelling is summarized in Table 3.

Raman spectra were collected using a Renishaw inVia (H43662 model, Gloucestershire, UK) equipped with a green laser line (514 nm) with a 50× objective. Raman spectra were recorded in the range from 250 cm^−1^ to 3500 cm^−1^. Decomposition of Raman spectra, the core of the present paper, was focused on the range 1000–2000 cm^−1^ and performed with a homemade software developed using Matlab^®^ (version R2020a).

X-ray diffraction (XRD) analyses were performed by using Panalytical X’PERT PRO PW3040/60 diffractometer, with Cu Kα radiation at 40 kV and 40 mA, Panalytical BV, Almelo, The Netherlands. The spectra were obtained from biochar powder in the 2θ range from 10 to 90° with a step size of 0.013°. For the decomposition of XRD peaks in the 2θ regions 15–35° and 40–50° two different software were employed in order to verify the reproducibility and consistency of the results: Panalytical HighScore Plus v3.0d and a homemade compiled software developed using Matlab^®^ (version R2020a).

In the XRD analysis, the two peaks under investigation, which were assigned to 002 and 100 reflection of graphite respectively, have been used to determine the degree of biochar crystallinity as a function of temperature. Three main parameters were calculated: d_002_ which represents the interlayers spacing of aromatic layers; L_a_ and L_c_ on their turn represent the in-plane size and stacking height (size along *c* axis) of crystallites respectively. The values of d_002_, L_a_ and L_c_ reported below are the mean values obtained using the two software previously mentioned.

The distance between graphitic layers was estimated using the Bragg equation:

*n* λ = 2 *d*_002_ sin(θ) (1)

where λ is the X-ray wavelength (0.154 nm as the Cu Kα radiation was used), and θ is the diffraction angle. L_a_ and L_c_ were calculated using Debye–Scherrer equation [23]

(2) $$L_{i}=\frac{K_{i}\lambda }{B_{i}\;cos\theta }$$

where *B_i_* is the full width at half maximum (FWHM) values of the relevant peak, *K_a_* is 1.84 and *K_c_* is 0.89.

## 3. Results

### 3.1. New Lineshape Definition: Theoretical Bacground and Mathematical Details

As the key point of this paper consists in the use of a peculiar lineshape for fitting biochar Raman spectra, before presenting and discussing the results it is worth to focus on the origin of such a lineshape. In a continuous wave excitation process the local equilibrium polarizability is perturbed by the external wave while it is restored by the decay processes. This leads to a memory effect that is mapped into the time correlation function (TCF) of the dipole moments [24]. Given a certain resonant vibration its lineshape and linewidth in the frequency domain are determined by the Fourier transform of such TCF [25]. The exact lineshape of the peak related to a given vibration in the Raman spectra is hence related to the mechanisms that dissipate the energy associated to the vibration mode activated by the interaction with the incoming photons. If a single decay mechanism is at work and all the oscillators are in the same environment the typical Debye relaxation occurs, characterized by a single lifetime leading to a Lorentzian shape in the Raman spectra [24]. On the other hand, if the local environment of the same oscillator type at different locations of the solid is different, the sum up of all contributions leads to a different lineshape. In solid state physics it is quite customary [26,27] to assume that this decay relaxation is of a Gaussian type, and its Fourier transform in the Raman spectrum has a Gaussian shape as well. However, this can be an oversimplification, depending on the type and amount of local disorder. Strictly speaking Gaussian distribution can occur only if the various lifetimes are independent while in a partially disordered or in an amorphous solid the situation can be trickier. 

**Figure 1:**
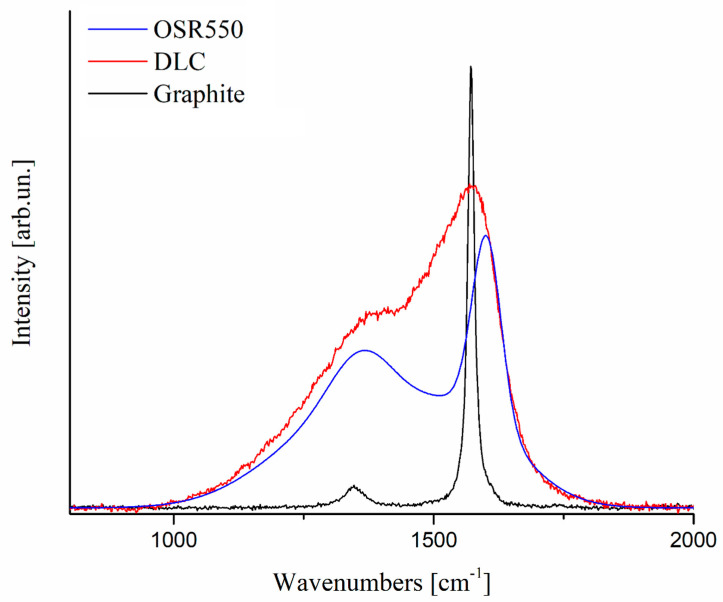
Overview on the lineshape of a highly ordered graphite (solid black), a Diamond-Like Carbon (DLC) (solid red) and oil seed rape (OSR) 550 (solid blue).

A quick look at Figure 1 where a typical Raman spectrum belonging to our set is compared to the spectrum of a highly disordered amorphous carbon (DLC: Diamond-Like Carbon) and of crystalline graphite shows that biochar, as well as many other carbon materials, such as carbon fibers, cannot be associated to either of them. As a consequence, while the use of Gaussian lineshapes for the D and G peaks is appropriate for DLC [5] and that of Lorentzian lineshape is appropriate for graphite our case is worth of a deeper discussion.

Lineshapes other than Lorentzian and Gaussian have been proposed and justified on physical grounds. As the basic phenomena occurs in the time domain it is worth to discuss decay processes in such a domain. Among the various types of relaxation, the stretched exponential one, besides being of interest in liquids [28] is also found quite often in disordered solids [29]. Hence, it appears a good candidate for our material too. The resulting TCF was first discussed by Rothschild, Perrot and Guillaume [30,31,32] and is appropriate to describe strongly interacting liquids [33,34] as well as disordered solids [35]. However, we must note that:

- we need to fit each Raman spectra in the frequency domain with a number of peaks by using a computational optimization algorithm;
- the Fourier transform of the TCF arising from the stretched exponential relaxation cannot be calculated in closed form;

These points will lead for every step of the optimization process to numerically compute the Fourier transform of all peaks for every frequency value. As a consequence, the computation time will be beyond any reasonable timescale for a typical PC or workstation.

Taking into account this limitation and also that:

- biochar materials appear to be only ‘partially’ disordered as shown in Figure 1
- the effect of disorder arises more strikingly at short and large times [36]

we introduce a specific lineshape in the frequency domain that is able to account for all physical facts although not being yet fully justified on theoretical grounds: a mixed Gaussian-Lorentzian shape (GauLor), symmetrical with respect to its central wavenumber *ω*_0_, in which the central part is Lorentzian and the tails are Gaussians. We apply the further constraint that the curve must be of class C^1^ (i.e., both the functions and the derivatives must be continuous) in the transition points. Being *ω_th,up_* (>*ω*_0_) the onset wavenumber of the upper Gaussian tail, each resonant peak is described by a two valued function:

(3) $$I_{Lor}\left(\omega \right)=\;I_{Lor_{\left(\omega_{0}\right)}}\;\frac{\Gamma /\pi }{4\;{\left(\omega -\omega_{0}\right)}^{2}+{\left(\Gamma \right)}^{2}}$$

(4) $$I_{Gau}\left(\omega \right)=I_{Gau_{\left(\omega_{0}\right)}}\;\frac{e^{-\frac{{\left(\omega \;-\;\omega_{0}\right)}^{2}}{2\;\sigma^{2}}}}{\sigma \;\sqrt{2\pi }}$$

where σ is the width of the Gaussian and *Γ* is the dissipation term of the Lorentzian. By applying the C^1^ constraint at *ω_th,up_* we have:

(5) $$\sigma =\sqrt{\frac{4{\left(\omega_{th,up}\;-\;\omega_{0}\right)}^{2}\;+\;\Gamma^{2}}{8}}$$

To summarize, our function is defined as follows:

(6) $$I_{\mathrm{GauLor}}(\omega )=\{\begin{array}{c} I_{Lor}\left(\omega \right)\;\;\mathrm{for}\;(\omega_{th,low}\leq \omega \leq \omega_{th.up})\\ I_{Gau}(\omega )\;\;\;\mathrm{for}\;\left(\omega \;\leq \omega_{th,low}\right)\;AND\;\left(\omega \;\geq \omega_{th,up}\right) \end{array}$$

where *ω_th,low_* is the threshold wavenumber for transition from Gaussian to Lorentzian shapes at wavenumbers in the low wavenumbers region. 

**Figure 2:**
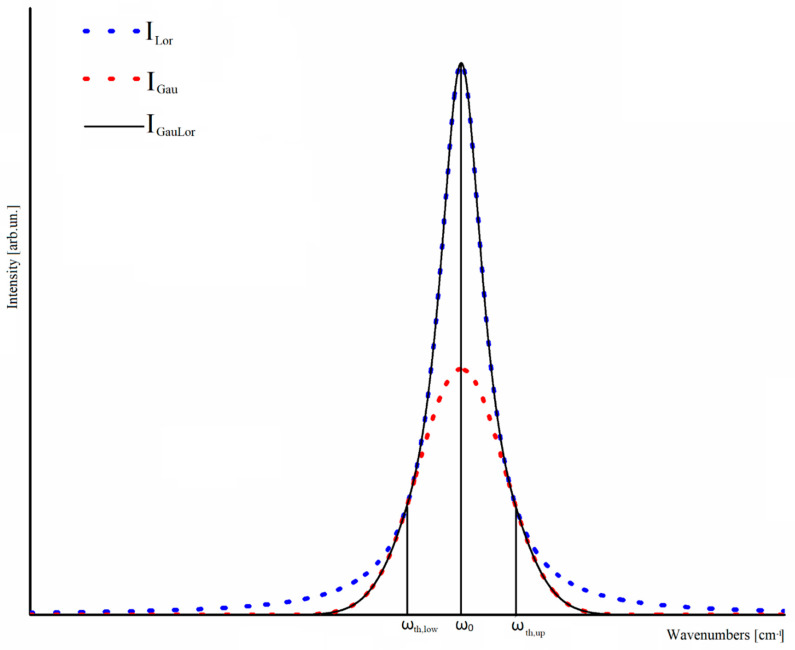
Superimposition of I_GauLor_ (solid black), I_Gau_ (**dotted red**) and I_Lor_ 550 (**dotted blue**).

A typical GauLor shape where the two transition points are symmetrical with respect to the peak wavenumber is reported in Figure 2.

### 3.2. Check Versus Stretched Exponential

As the stretched exponential is the most meaningful time decay in disordered materials, the first test for our GauLor is to check if it is mimicking well enough the Fourier transform arising from the TCF of a stretched exponential decay, that is described by:

(7) $$I\left(t\right)=I_{0}\;e^{-{\left(\frac{t}{\tau }\right)}^{\beta }}$$

where τ is the relaxation time and β is a positive number lower than 1. Hence, we proceed as follows:

- we fix a set of parameters for a stretched exponential relaxation (β = 0.8)
- we compute the TCF Gν(t) by using equation [25]
  (8) $$-\frac{\ln\left(G_{\nu }\left(t\right)\right)}{M_{2}\;\tau^{2}}=\overset{\infty }{\underset{n=0}{\sum }}\frac{{\left(-1\right)}^{n}{\left(t/\tau \right)}^{2+n\beta }}{n!\left(1+n\;\beta \right)\left(2+n\;\beta \right)}$$
  where *M*_2_ (a constant value for a given peak) is the vibrational second moment [24]
- we compute the TCF Fourier transform numerically for each wavenumber
- we fit this Fourier transform with a single GauLor selecting its parameters with the fitting procedure detailed in the next paragraph

**Figure 3:**
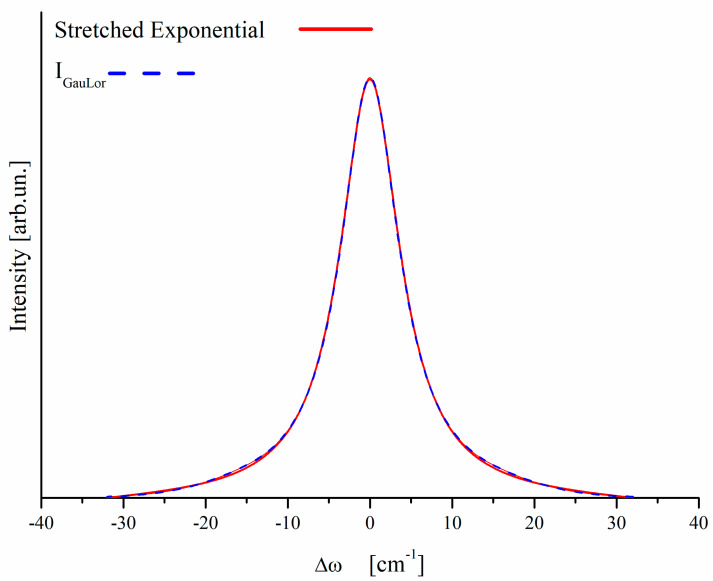
Superimposition of I_GauLor_ (**dotted blue**) and stretched exponential (**solid red**).

The results of this procedure are shown in Figure 3. We clearly see that:

- the stretched exponential based Raman peak is symmetrical with respect to its peak wavenumber [28],
- the GauLor curve mimics very well the stretched exponential based peak,

Taking into account (i) the physical significance of stretched relaxation for disordered materials, (ii) the symmetry of the Raman peak arising from it, and (iii) the excellent mimicking that GauLor provides for the stretched exponential based Raman peak, from now on we confidently use GauLor shapes to fit Raman spectra, assuming for each of them that the two transition points are symmetrical with respect to the peak position.

### 3.3. Details of the Fitting Procedure and Evaluation of the Contribution of the Lorentzian and Gaussian Parts to the Overall Intensity of a Peak

Given that the recorded Raman spectra are discrete and not continuous we have M wavenumber values in the wavenumber range of interest for the fit. For each wavenumber *ω_i_* the measured signal has an intensity S(*ω_i_*). 

As a first step we define, on meaningful physical basis, the number N of GauLor components we use for the spectra decomposition. For each component we have to give initial tentative values to the following parameters: *ω*_0_, *ω_th,up_*, σ. 

For each wavenumber *ω_i_* we compute the fitting curve intensity as follows

(9) $$S_{Fit}\left(\omega_{i}\right)=\sum_{j=1}^{N}I_{GauLor}\left(\omega_{i},\omega_{0,j},\;\omega_{th,up,j},\sigma_{j}\right)$$

The optimization algorithm targets the maximum similarity between the measured and fitting curves. Such similarity is obtained applying the Levenberg–Marquardt algorithm [37] targeting the minimization parameter defined as:

(10) $$Χ=\sum_{i=1}^{M}{\left[S\left(\omega_{i}\right)-S_{Fit}\left(\omega_{i}\right)\right]}^{2}$$

The algorithm is run by varying the *ω*_0_, *ω_th,up_*, *σ* parameters for each GauLor until the value of X for two subsequent optimizing steps is below a given threshold.

As the role of disorder is to lead to a departure from the Lorentzian shape it is worthy to evaluate the relative contributions of Gaussian and Lorentzian parts to each GauLor. To this purpose we define $I_{Lor}^{tot}\;$ as the total area of the GauLor between *ω_th,low_* and *ω_th,up_* where the GauLor has a lorentzian shape:

(11) $$I_{Lor}^{tot}=\int_{\omega_{th,low}}^{\omega_{th,up}}I_{GauLor}\left(\omega ,\omega_{0},\;\omega_{th,up},\sigma \right)d\omega$$

By computing as well the total area $I_{GauLor}^{tot}$ under the GauLor peak:

(12) $$I_{GauLor}^{tot}=\int_{-\infty }^{+\infty }I_{GauLor}\left(\omega ,\omega_{0},\;\omega_{th,up},\sigma \right)d\omega$$

we are able to evaluate the fraction of the GauLor area described by its Lorentzian shaped part:

(13) $$f_{L}=\frac{I_{Lor}^{tot}}{I_{GauLor}^{tot}}$$

The fraction of the area for the Gaussian part is:

(14) $$f_{G}=1-f_{L}$$

### 3.4. Approach to the Fit of the Set of Samples

As the Raman signal of OSR 2200 show well resolved and sharp peaks (see below) we started by fitting it as the uniqueness of the fit is better guaranteed. With the set of GauLor components obtained we fitted the Raman spectra of all other samples. Any additional peak needed to fit such spectra with respect to OSR 2200 was justified on physical basis. 

### 3.5. XRD Analysis

In order to better understand and support the results of our Raman analysis we have performed a thorough XRD analysis with the purpose to investigate the evolution of the nanographitc domains of biochar structure.

**Figure 4:**
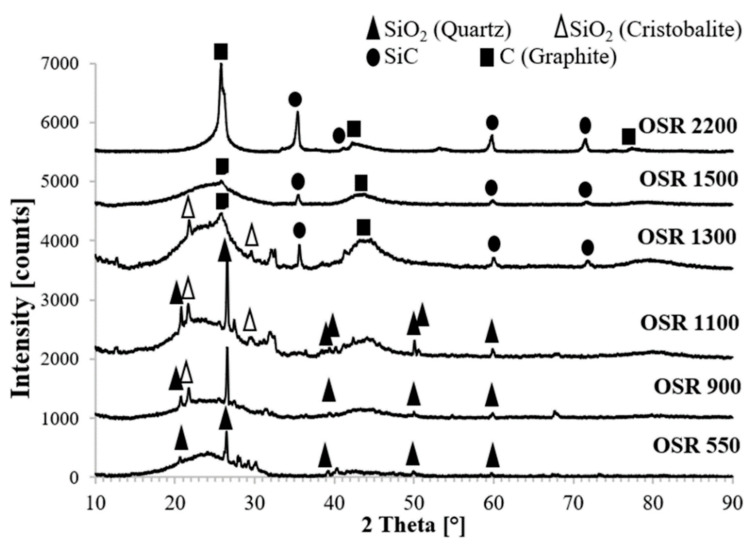
XRD patterns of biochar after thermal treatment at the various temperatures.

Figure 4 compares the X-ray diffraction patterns of biochar coming from oilseed rape after thermal treatment at increasing temperature from 550 °C to 2200 °C. Sample OSR 550 shows the presence of SiO_2_ in quartz form as the main crystalline phase; starting from OSR 900 cristobalite is present as well. Silica is a common component of biochar especially when it comes from grass, straw and grain husks [38]. Some low intense peaks appear in both the spectra at 2θ~21.8°, 28.0°, 29.3° and 30.2°. The presence of few peaks with very low intensity make difficult to state to which crystalline phase they belong. However, it appears that they cannot be assigned to a single compound. EDS analysis performed on OSR 550 [20] revealed the presence of magnesium, potassium, calcium, sulfur, silicon and aluminum as inorganic components. Moreover, the 2θ position of the peaks under investigation is consistent with the presence of Mg–Al silicate, CaCO_3_ and K_2_SO_4_. Therefore, we can hypothesize that these low intense peaks come from salts present as inorganic components of original biomass. This is in agreement with the literature: Xu et al. [39] reported the presence of a fraction of mineral compounds such as alkali metals or alkaline earth metals in form of carbonates, phosphates or oxides in many kinds of biochar, in addition to the carbon main component. At higher temperature these peaks disappeared probably due to thermal degradation phenomena. 

The XRD patterns from OSR 550 to OSR 1500 show a broad hump in the 2θ region from 16° to 32° indicating that biochar contains a portion of highly disordered material in the form of amorphous carbon. When the treatment temperature reaches 1500 °C, a peak at 2θ~26°, characteristics of (002) graphite reflection, emerged from the hump.

The asymmetry of this band can be explained by the presence of a second peak at lower angle. Some authors [40,41,42] fitted the broad hump using two Gaussian curves centered at 20° and 25.5° respectively. The first peak called γ-band can be attributed to saturated structures such as aliphatic components linked to char crystallites edges. The second П-band at 2θ~25.5° can be attributed to the spacing between aromatic ring layers [43]. Moreover, the XRD spectrum of OSR 1500 shows (100) and (110) reflections of graphite at 2θ~43° and 77° respectively; these peaks are also present in OSR 550 and OSR 900 spectra, although with lower intensity and width. The presence of these more defined peaks confirms the existence of crystallites in the treated biochar [43] and their broad shape suggests a very small size of the formed crystallites. These observations suggest that the treatment at 1500 °C leads to an evolution of biochar carbon fraction in a structure which is intermediate between amorphous and the graphitic turbostratic structure [44]. 

A further increment of pyrolysis temperature up to 2200 °C led to the disappearance of the broad hump; the presence of sharper and more defined peaks clearly indicates a more crystalline structure of biochar. The turbostratic structure seems indeed to further evolve in a more ordered graphitic one whose presence is confirmed by a defined peak at 2θ~26.2°. The less intense peaks at 2θ~43° and 77° correspond to (100) and (110) reflections of graphite as stated above. These previously observed peaks become more shaped and defined when treatment temperature reached 2200 °C. 

In addition, the spectra of OSR 1300, OSR 1500 and OSR 2200 show the presence of 3C-SiC phase coming from the reaction between SiO_2_ and carbon. The presence of β-SiC can be justified considering that the main commercial process for its production involves the carbothermal reduction of silica using different kind of carbonaceous materials such as petroleum coke, anthracite and bituminous coals or carbon black. The process is known as Acheson process [45]; it involves temperatures in excess of 2000 °C and it is strongly dependent both on temperature and gas composition. According to Kurunov et al. [46] the initial temperature for the reaction between silica and carbon ranges from 1200 to 1350 °C. Li et al. [47] reported the presence of a weak peak of β-SiC starting from 1300 °C for samples containing a mixture of silica and graphite treated in argon atmosphere. This justifies the presence of traces of SiC from OSR 1300 sample; it shows a cubic structure which is the most common polytype formed during the carbothermal reduction process.

**Figure 5:**
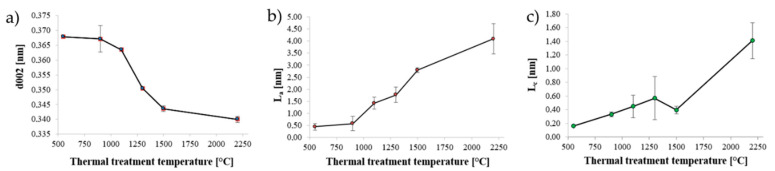
Variation of crystallites parameters as function of thermal treatment temperatures: (**a**) interlayers spacing of aromatic layers, (**b**) in-plane size and (**c**) stacking height of crystallites.

Figure 5 shows the trend of the three parameters d_002_, L_a_ and L_c_, which are representative of the average size of graphitic crystallites, as a function of the increasing thermal treatment temperatures. 

Figure 5a reveals similar values of interlayers spacing after treatment at 550 °C and 900 °C which were 0.368 nm and 0.367 nm respectively. After thermal treatment at 1500 °C and 2200 °C, d_002_ steeply decreases reaching values of 0.344 nm and 0.340 nm, which are closer to the interlayer spacing of graphite (0.335 nm as reported by Jeong et al. [48]. Figure 5b,c reveal that the size of crystallites progressively increases with the increasing of treatment temperature: the in-plane size from around 0.40 nm for OSR 550 to 4.09 nm for OSR 2200. The crystallites size along *c* axis increases from 0.16 nm for OSR 550 to 1.41 nm for OSR 2200.

Furthermore, XRD analysis represent a powerful tool to evaluate the raising of SiC during the increment of treatment temperature. This evidence will be usefully in the next section for the explanation of Gaussian fraction (f_G_) trend.

### 3.6. Raman Spectra Analysis

**Figure 6:**
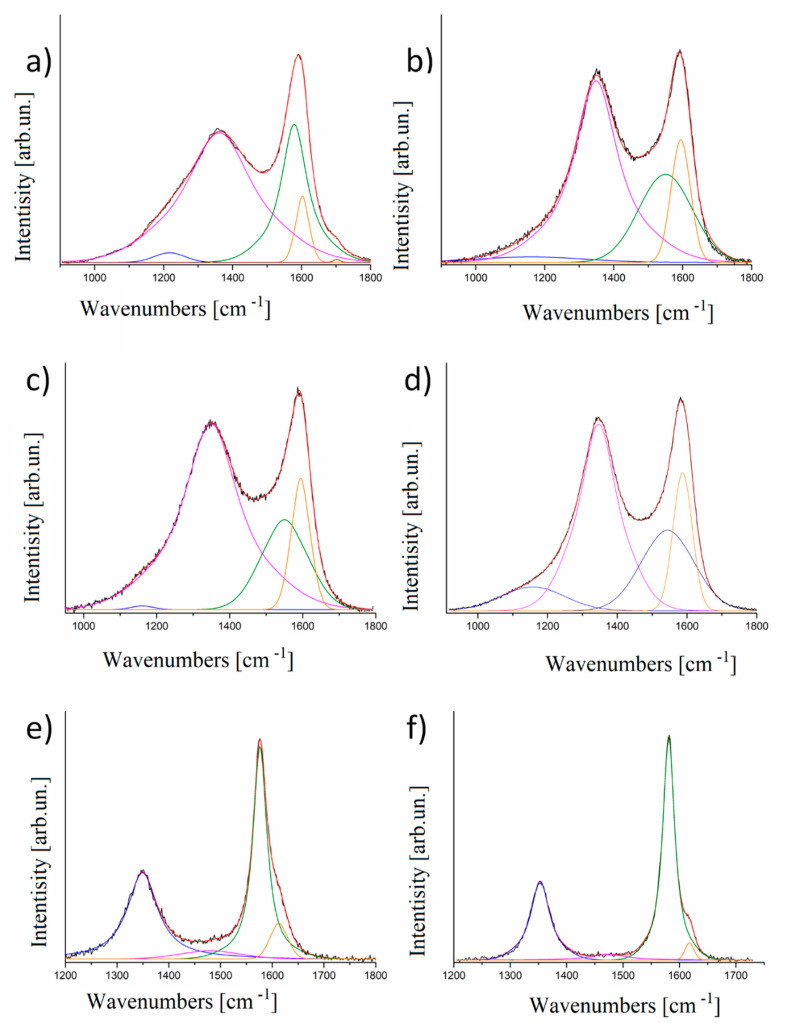
Raman spectra and related fit using I_GauLor_ of (**a**) OSR550, (**b**) OSR900, (**c**) OSR1100, (**d**) OSR 1300, (**e**) OSR1500 and (**f**) OSR 2200. Original Raman spectra are reported in black, the fitted Raman spectra are reported in red.

In Figure 6 we report the Raman spectra measured for the set of thermally treated samples. Spectra have been normalized to the height of the right peak in order to render features more evident.

The spectra suggest that there is an evolution related to the treatment temperature and clearly evidence the correlation among the spectra obtained for the biochars treated at the various temperatures. In Figure 6f the Raman spectrum of OSR2200 is reported together with the fitting obtained using two main $I_{\mathrm{GauLor}}$ shaped peaks, one (left–lower wavenumbers named D^1^ or D^1^) associated to the D-mode and the other (right–higher wavenumbers G^1^) associated to the G-mode. Two additional peaks are present, one at low wavenumber and one at high wavenumber namely respectively D^2^ and G^2^ (particularly evident in OSR 2200,) on which we will comment in detail later. The same set of GauLor was used to satisfactorily fit the Raman spectra obtained for most other temperatures (Figure 6b–e). As a first comment we note that our approach appears to be consistent in analyzing the various spectra as well as based on sound physical grounds as:

- it is based on two well assessed peaks for carbon sp^2^ coordinated materials;
- it takes into account the peculiarity of the impact of structural disorder and TCF on Raman spectra.

If we focus on the evolution of the signal for increasing temperature considering the thermal evolution of the material, we notice that the D-peak and the G-peak both become narrower.

D peak arises from aromatic rings confined in defective graphitic structures and it is related to the size of crystallites [49] and their edges [50], while G peak is related to in-plane carbon sp^2^ bonds stretching. 

**Figure 7:**
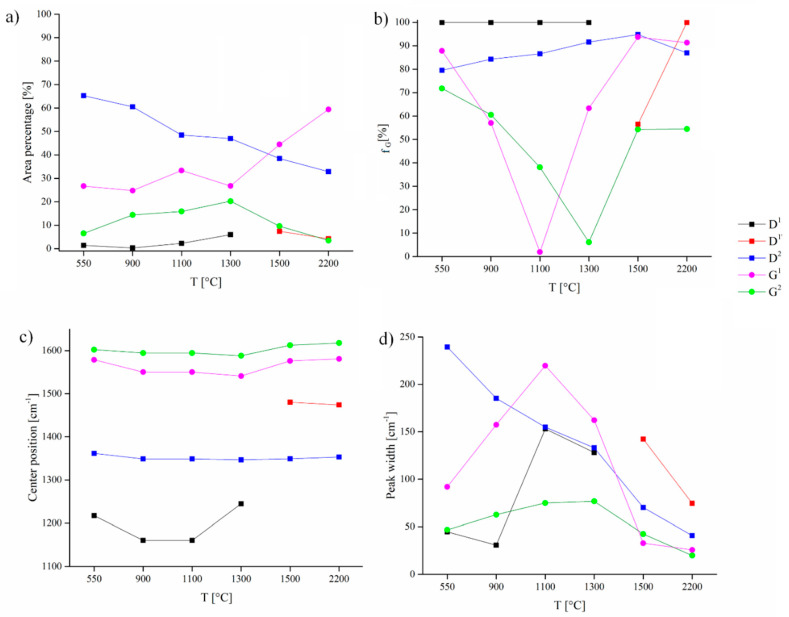
Components trends for (**a**) area percentage, (**b**) f_G_, (**c**) peaks centers and (**d**) widths.

This assignation is supported by the trend vs treatment temperature of the various features of the spectra and of the GauLor components. Figure 7 shows the trends of the more relevant parameters as a function of the carbonization temperature.

D and G peaks were fitted by only two components for temperatures with the exception of OSR 550 where an additional component centered at 1702.2 cm^−1^ was added. This component has a very low intensity with a relative area percentage of 0.1 % and could be attributed to inorganic residues. Tilli et al. [51] attributed to CaCO_3_·nH_2_O active Raman modes ranging from 1738 cm^−1^ to 1746 cm^−1^. In our specific case, OSR 550 contains calcium and it is reasonable to assume that the calcium carbonates residues embedded into a carbon matrix could have low Raman frequencies due to the local carbonaceous environment. Furthermore, this component disappeared at higher temperature according to degradation of carbonates at temperature beyond 900 °C [52] and it was not detectable by XRD due the low amount of the species. 

**Figure 8:**
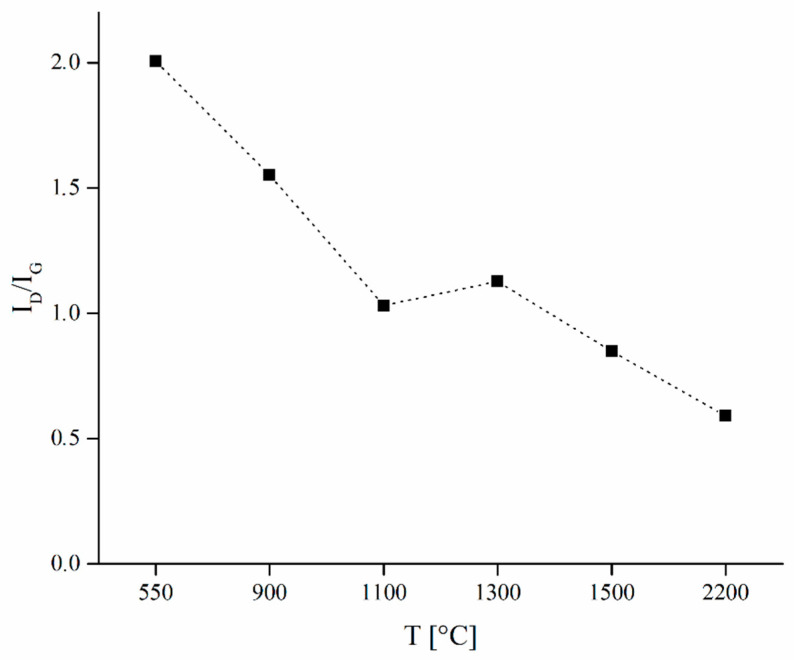
Trend between I_D_/I_G_ ratio and carbonization temperature of OSR samples.

Comparing Figure 5 and Figure 7, G^1^ peak appears to be proportional to reduction of interlayers spacing of aromatic layers and increment of graphitic crystallites showing an origin related to the ordering of the carbonaceous material. G^2^ area percentage trend was more complex showing a constant increment of both area percentage and band width of up to 1300 °C and a decrement afterwards. We hypothesized G^2^ was related with bond angle disorder as suggest by Shimodaira et al. [53]. Considering graphitic turbostratic rearrangement, it is reasonable to assume a massive layer stacking driven by π–π orbital alignment when the temperature threshold was reached inducing a short-range order. We located this transition at 1300 °C when we observed a decrement of G^2^ area percentage due to the reorganization of carbon structures [54]. Both G^1^ and G^2^ peak positions were quite stable, being centered respectively at 1563 ± 17 cm^−1^ and 1601 ± 11 cm^−1^. D^2^ peak showed an opposite trend compared to G^1^ peak and we assumed it to be related to the presence of confined regions of small size as reported by Negri et al. [50]. The interpretation of the other D components was more complex and we hypothesized that they rose from two different but similar phenomena. According to Shimodaira et al. [53], the components named D^1^ and D^1′^ are both related to angle disorder but we advance the hypothesis that they were due by graphitic domains misalignment induced by inorganic components. Inorganics represent of up to 19.5 wt.% of OSR 550 [20] and are uniformly dispersed in the carbon matrix. When sp^2^ clusters started to grow inorganics were excluded from the same area inducing at same time a stress on the aromatic domains. This effect generated the D^1^ component, replaced by D^1′^ above 1300 °C. At a temperature higher than 1300 °C in fact, graphitic crystallites size rapidly grew simultaneously to the formation of SiC though carbothermal processes. In this environment, SiC region embedded into aromatic domains can perturb the bond angles of carbon planes originating the band D^1′^ that show a peak center position at higher frequencies than D^1^ (Figure 7c). The disorder related to D^1^ and D^1′^ was attested by f_G_ values of 100% for D^1^ close to 90% for D^1′^. Similarly, D^2^ show a constant increment in f_G_ attesting a disordering phenomenon. The f_G_ trends of G^1^ and G^2^ were more complex showing a decrement with the increment of temperature up to 1100–1300 °C and increment with further temperature increase. These observations witness the presence of inclusion of SiC into graphitic domains formed at high temperature with a consequently Gaussian-like disordered phenomena. As clearly emerged, 1300 °C is a key temperature for the thermal evolution of the OSR to a more ordered state. This was supported also by the evaluation of I_D_/I_G_ ratio reported in Figure 8.

I_D_/I_G_ ratio was calculated as a ratio between the total area of the D and of the G components. It constantly decreased up to and beyond 1300 °C while between 1100 °C and 1300 °C increased. This behaviour is partially in agreement with Ferrari et al. [5] that reported an increment of I_D_/I_G_ ratio following the transition from amorphous carbon to nanocrystalline graphite. In our specific case, we observed an additional phenomenon of ordering due to the degradation of all non-carbonaceous structure together with the clustering of the *sp*^2^ phase and evolution of inorganic matters with the formation of SiC. Infiltration of SiC into nanographitic domains was detailed described by Padovano et al. [55] proving its incorporation into the a carbon structure. The combination of the processes above could affect the D evolution creating more defects than in the case of the carbonization of pure carbon film generating the complex trend observed for f_G_.

We observed high values of f_G_ for both components of D peak (Figure 5b) while the f_G_ components of G peak decreased up to 1300 °C and increased afterwards. This could be explained by the raising of SiC formation, reported also by XRD analysis, that slow down the ordering process of graphitic crystallites. Centers of the components increased alongside all temperature range according with literature [5,56] while components width increased up to 1300 °C and decreased afterwards.

These trends confirmed the reorganization of the OSR with increasing of the carbonization temperature accordingly with XRD data from Figure 6. Both techniques proved that the material is still reorganizing itself to graphitic structure. This process cannot complete in biochar samples considered in this paper due to the presence of inorganic species as detected by XRD and also because of the natural porous structure of the material. These two factors are responsible for the somewhat hindered reorganization of nanographitic domains. 

**Figure 9:**
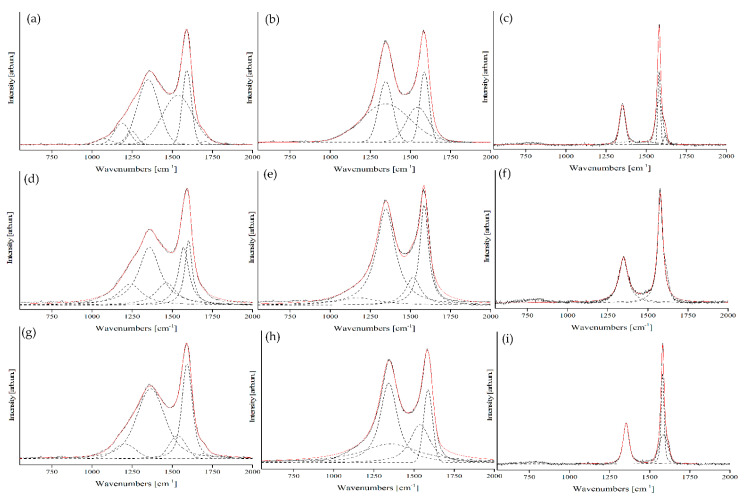
Raman spectra and related fit using I_Gau_ (**a**–**c**), I_Lor_ (**d**–**f**) and Voigt functions (**g**–**i**) of OSR 550, OSR 1300 and OSR 2200 respectively. Original Raman spectra are reported by using solid black lines, the fitted Raman spectra are reported by using solid red lines and each component is reported in dashed black lines.

For a strong validation of proposed fitting methodology, we evaluated the fit of Raman spectra by using I_Gau,_ I_Lor_ and Voigt functions for each sample (Supplementary Materials) and we reported the most significant in Figure 9.

Fit of Raman spectra by using I_Gau_ (Figure 9a–c) required a high number of components of up to seven for the OSR 550 spectrum, up to five for OSR 900 and four for OSR 1100, OSR 1300 and OSR 1500 (Supplementary Materials, Figure S1). Spectrum of OSR 2200 (Figure 9c) required only three components but due to the particular shape of the I_G_ peak two of them are concentric. This mathematical fitting was quite accurate but lacked physical ground and failed to explained the mode that originated the components.

Fit of Raman spectra by using I_Lor_ were challenging. Fit of OSR 550 (Figure 9d) and OSR 900 (Supplementary Materials, Figure S1) and required respectively five and four components. The fit OSR carbonized at 1100 °C–1500 °C degrees showed some problem related to I_Lor_ tails drop (see supporting information and Figure 9e for OSR1300). The line shape of did not allow a correct fit (Figure 9k,n,q) of I_D_ and I_G_ peaks with an overestimation of their area. Nonetheless, I_Lor_ fitted correctly the centered region of the I_D_ and I_G_ peaks of materials treated in a temperature range from 1100 °C up to 2200 °C. 

Voigt functions results were comparable with I_GauLor_ for OSR 550 (Figure 9g), OSR 1100 and OSR 1300 (Supplementary Materials, Figure S1). Nonetheless, they failed to produce an accurate fit for OSR 1300 (Figure 9h) and they showed the same problem of I_Gau_ in the fitting of OSR 2200 (Figure 9i) with the two components of I_G_ peaks that were practically concentric. Fit with pseudo-Voigt lead results to the presence of components without any physical features (i.e., multimodal or negative curves) that could eventually (Supplementary Materials Figure S2).

From data reported in Figure 9, it was arguable that traditional established lineshapes cannot provide a solid base for an accurate fitting on a wide range of carbonization temperature contrary to I_GauLor_ that showed a constant number of components with very accurate fit.

Moreover, it has to be noted that the peak position in the case of Voigt and Gaussian shapes do not move with a clear trend with treatment temperature.

## 4. Conclusions

In this paper, we successfully propose and validate a ground-breaking approach to Raman signal processing of disordered and nanostructured carbons. The GauLor lineshape introduced for the Raman fit is supported both by numerical accuracy and a valid physics explanation. To test this new methodology, Biochar was investigated as a complex case study due the massive presence of inorganics. These components deeply affected the carbonization process as clearly showed by the XRD analysis that enlighten the formation of SiC inclusion into carbonaceous structure. Due its composition and hierarchical structure, biochar Raman signal processing represent a challenging bench test for our new methodology. The biochar Raman signal was successfully fitted using this innovative GauLor lineshape and it was proved as a reasonable approach to describe complex carbon materials by comparing it with established methodologies. The procedure can also be used for all real carbon samples as it promotes a more accurate decomposition of Raman spectra.

## Figures and Tables

**Table 1:** Main chemical and physical properties of biochar samples as reported by safety data sheets [21].

4	Proximate Analysis [wt%]				Ultimate Analysis [%]			Specific Surface Area [m^2^/g]
	Moisture	Ash	VOCs	Fixed Carbon ^a^	C	H	O ^b^	
OSR 550	2.61	19.50	16.38	61.51	68.85	1.82	8.91	7.3

^a^ Calculated as difference, ^b^ calculated as difference considering the ash content.

**Table 2:** Elemental composition of OSR 550 as emerged by energy dispersive X-ray detector (EDX).

Element	Concentration [wt.%]		
	Site 1	Site 2	Site 3
C	86.79	90.67	34.45
O	10.82	8.49	42.33
Mg	0.10	0.05	0.29
Si	0.33	0.15	0.41
P	0.12	0.05	0.16
Cl	0.10	0.03	1.88
K	1.32	0.42	20.48
Ca	0.42	0.13	34.45

**Table 3:** Samples labelling.

Samples Label.	Treatment Temperature [°C]
OSR 550	550 ^a^
OSR 900	900 ^b^
OSR 1000	1000 ^b^
OSR 1100	1100 ^b^
OSR 1300	1300 ^b^
OSR 1500	1500 ^b^
OSR 2200	2200 ^b^

^a^ pyrolysis process, ^b^ carbonization process.

## Supplementary Materials

The following are available online at https://www.mdpi.com/2079-4991/10/9/1748/s1. Figure S1: Raman spectra and related fit using respectively IGau, ILor and Voigt functions of (a–c) OSR550, (d–f) OSR900, (g–i) OSR1100, (j–l) OSR 1300, (m–o) OSR1500 and (p–r) OSR 2200., Figure S2: Raman spectra and related fit using respectively pseudo-Voigt functions of (a) OSR550, (b) OSR900, (c) OSR1100, (d) OSR 1300, (e) OSR1500 and (f) OSR 2200
