# Supplementary material for: Introducing the Novel Mixed Gaussian-Lorentzian Lineshape in the Analysis of the Raman Signal of Biochar
Source: Nanomaterials (Basel). 2020 Sep 3;10(9):1748. doi: 10.3390/nano10091748 (PMC7558824; doi:10.3390/nano10091748)
Supplement: Supplementary file 1 [file nanomaterials-10-01748-s001.pdf]

nanomaterials

MDPI

Article

Introducing the Novel Mixed Gaussian-Lorentzian Lineshape in the Analysis of the Raman Signal of Biochar

Alberto Tagliaferro [1,2,3,*], Massimo Rovere [1,3], Elisa Padovano [1], Mattia Bartoli [1,3] and Mauro Giorcelli [1,3]

1 Department of Applied Science and Technology, Politecnico di Torino, Corso Duca degli Abruzzi 24, 10129 Turin, Italy; massimo.rovere@polito.it (M.R.); elisa.padovano@polito.it (E.P.); mattia.bartoli@polito.it (M.B.); mauro.giorcelli@polito.it (M.G.)

2 Faculty of Science, Ontario Tech University, 2000 Simcoe Street North, Oshawa L1G 0C5 T, Ontario, Canada

3 Consorzio Interuniversitario Nazionale per la Scienza e Tecnologia dei Materiali (INSTM), Via G. Giusti 9, 50121 Florence, Italy

* Correspondence: alberto.tagliaferro@polito.it (A.T.); Tel.: +39-011-0907347

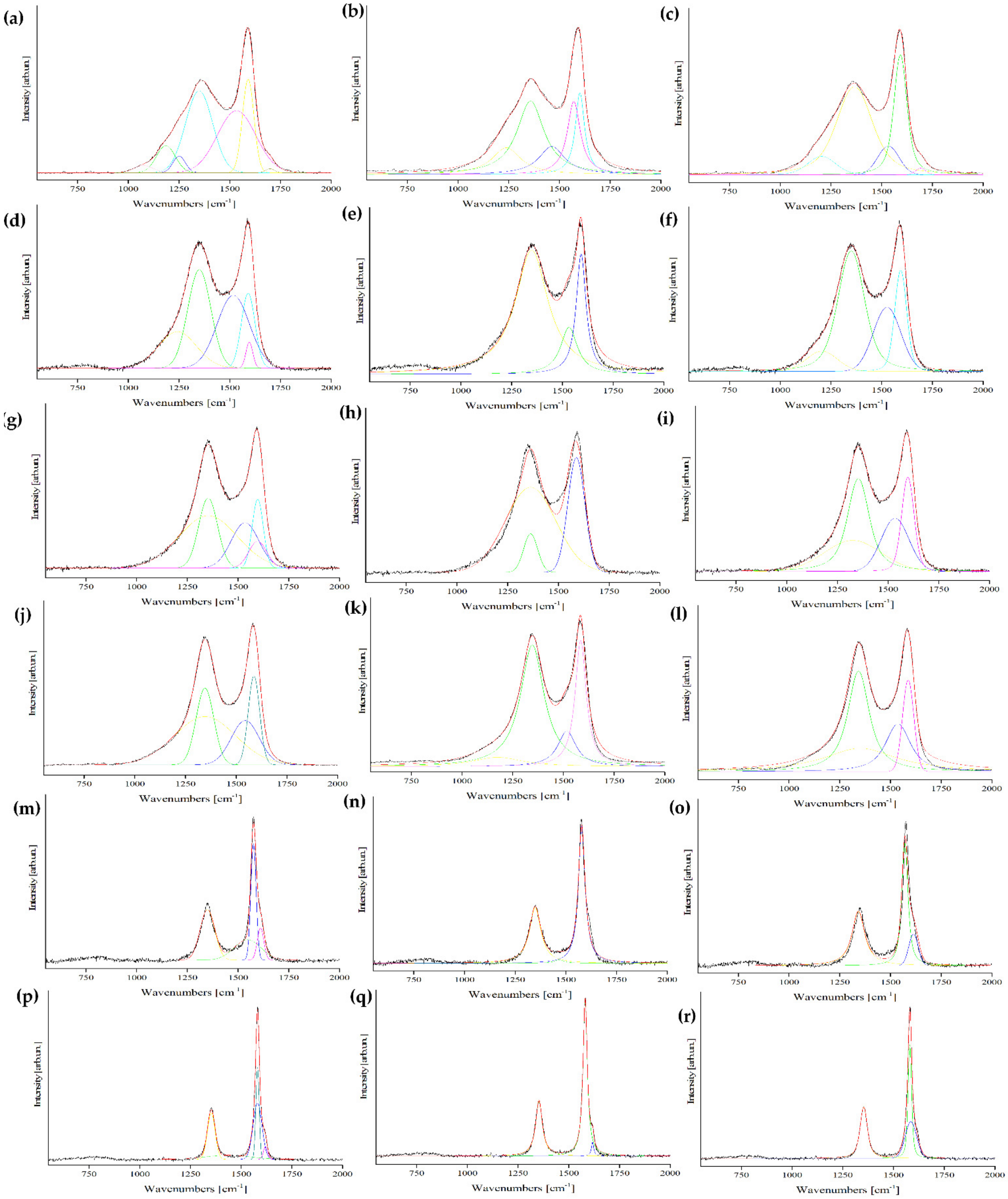

Figure S1. Raman spectra and related fit using respectively I_{Gau}, I_{Lor} and Voigt functions of a-c) OSR550, d-f) OSR900, g-i) OSR1100, j-l) OSR 1300, m-o) OSR1500 and p-r) OSR 2200. Original Raman spectra are reported in black, the fitted Raman spectra are reported in red.

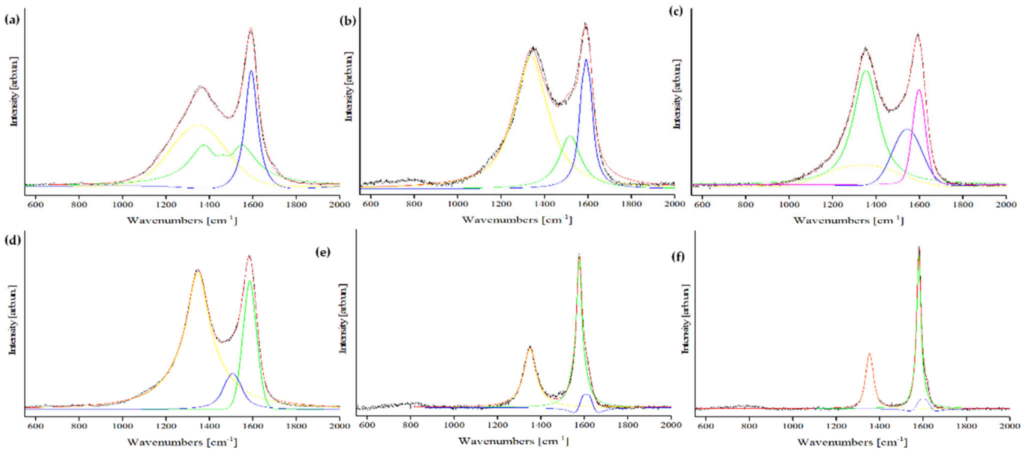

Figure S2. Raman spectra and related fit using respectively pseudo-Voigt functions of a) OSR550, b) OSR900, c) OSR1100, d) OSR 1300, e) OSR1500 and f) OSR 2200. Original Raman spectra are reported in black, the fitted Raman spectra are reported in red.
